# Evolution, structure and emerging roles of C1ORF112 in DNA replication, DNA damage responses, and cancer

**DOI:** 10.1007/s00018-021-03789-8

**Published:** 2021-02-24

**Authors:** Jacob Edogbanya, Daniela Tejada‐Martinez, Nigel J. Jones, Amit Jaiswal, Sarah Bell, Rui Cordeiro, Sipko van Dam, Daniel J. Rigden, João Pedro de Magalhães

**Affiliations:** 1 Integrative Genomics of Ageing Group, Institute of Ageing and Chronic Disease, University of Liverpool, Liverpool, L7 8TX UK; 2 Programa de Doctorado en Ciencias mención Ecología Y Evolución, Facultad de Ciencias, Instituto de Ciencias Ambientales Y Evolutivas, Universidad Austral de Chile, Valdivia, 5090000 Chile; 3 Department of Biochemistry and Molecular Biology, Thomas Jefferson University, Philadelphia, PA 19107 USA; 4 Institute of Systems, Molecular and Integrative Biology, University of Liverpool, Liverpool, L69 7ZB UK; 5 Institute of Aging Research, School of Medicine, Hangzhou Normal University, Hangzhou, China; 6 Department of Endocrinology, University of Groningen, University Medical Center Groningen, Hanzeplein 1, 9713 GZ Groningen, The Netherlands; 7 Ancora Health, Herestraat 106, 9711 LM Groningen, The Netherlands; 8 Faculty of Biological Sciences, Friedrich Schiller University, Jena, Germany

**Keywords:** BC055324, DNA repair, Oncogene, Tumour, Fanconi anaemia

## Abstract

The *C1ORF112* gene initially drew attention when it was found to be strongly co‐expressed with several genes previously associated with cancer and implicated in DNA repair and cell cycle regulation, such as *RAD51* and the *BRCA* genes. The molecular functions of C1ORF112 remain poorly understood, yet several studies have uncovered clues as to its potential functions. Here, we review the current knowledge on C1ORF112 biology, its evolutionary history, possible functions, and its potential relevance to cancer. C1ORF112 is conserved throughout eukaryotes, from plants to humans, and is very highly conserved in primates. Protein models suggest that C1ORF112 is an alpha-helical protein. Interestingly, homozygous knockout mice are not viable, suggesting an essential role for C1ORF112 in mammalian development. Gene expression data show that, among human tissues, *C1ORF112* is highly expressed in the testes and overexpressed in various cancers when compared to healthy tissues. *C1ORF112* has also been shown to have altered levels of expression in some tumours with mutant TP53. Recent screens associate C1ORF112 with DNA replication and reveal possible links to DNA damage repair pathways, including the Fanconi anaemia pathway and homologous recombination. These insights provide important avenues for future research in our efforts to understand the functions and potential disease relevance of C1ORF112.

## Introduction

Genomic and proteomic technologies have facilitated the rapid generation and analysis of large volumes of data [1, 2]. In turn, this has enabled researchers to discover and characterise novel genes to further our understanding of the role of cellular networks and pathways within biological and disease processes. In one such high‐throughput analysis, van Dam et al. (2012) identified the mouse *BC055324* gene [3], whose human ortholog is *C1ORF112*, as being strongly co‐expressed with genes previously associated with cancer in the literature, like *RAD51* and *CCDC6*. C1ORF112 is also co-expressed with many genes in the BRCA–Fanconi anaemia-related DNA damage response pathway, including *BRCA1*, *BRCA2*, *FANCD2*, and *FANCI* [3]. Defects in this pathway are associated with increased cancer risk [4].

The breast cancer susceptibility proteins BRCA1 (also known as FANCS), BRCA2 (FANCD1), and RAD51 (FANCR) and its paralogs, including XRCC2 (FANCU) and XRCC3, all serve a function in homologous recombination repair (HRR). HRR is a critical DNA repair process which operates not only in addressing directly occurring DNA double‐strand breaks, but also in the repair of broken and stalled DNA replication forks [5]. FANCD2 and FANCI are critical proteins that are mono‐ubiquitylated as part of the activation process within the Fanconi anaemia (FA) pathway, which is required for the repair of inter‐strand crosslinks (ICLs) [6]. Individuals with defects in the FA pathway are highly cancer-prone and are particularly susceptible to acute myeloid leukaemia and head and neck squamous cell carcinoma [4]. FANCD2 and FANCI lie at the centre of the FA‐BRCA pathway and are mono‐ubiquitylated by the upstream FA core complex (comprising nine FA or FA‐associated proteins). Downstream of the mono‐ubiquitylation of FANCD2 and FANCI are the FA proteins that function directly in DNA repair, including HRR. ICLs are a specific form of DNA damage that block transcription and DNA replication, and require removal by several DNA repair processes, including translesion DNA synthesis and HRR, which are in turn coordinated by the FA pathway. Whilst repair by HRR is largely error‐free when it, or the FA pathway itself, is defective; DNA double‐strand breaks and broken replication forks may be erroneously repaired by non‐homologous end joining (NHEJ) [7]. In *Arabidopsis thaliana*, a role for *C1ORF112* has been implicated in all these DNA damage response processes [8]. Earlier studies hinted of a possible association of C1ORF112 with cancer [9, 10]. For example, in a study of bladder cancer progression, the genomic and proteomic profiles in association with TP53 show that *C1ORF112* has a gene expression fold change corresponding to an increased expression with tumours having mutant *TP53*, an oncogene involved in driving various cancers [9]. Another study concerning gene expression in response to regulation by a progesterone hormone‐dependent breast cancer transfected with progesterone receptors exhibited a down‐regulation of *C1ORF112* expression, suggesting that *C1ORF112* might be a target for progesterone regulation [10].

Considering this background, we suggest that *C1ORF112* could be a gene of significant biological and clinical interest. Here, we review what we know about C1ORF112, discuss its potential functions, identify possible cellular pathways in which it may operate, and ascribe possible roles in diseases such as cancer.

### Sequence characteristics of C1ORF112

**Fig. 1:**
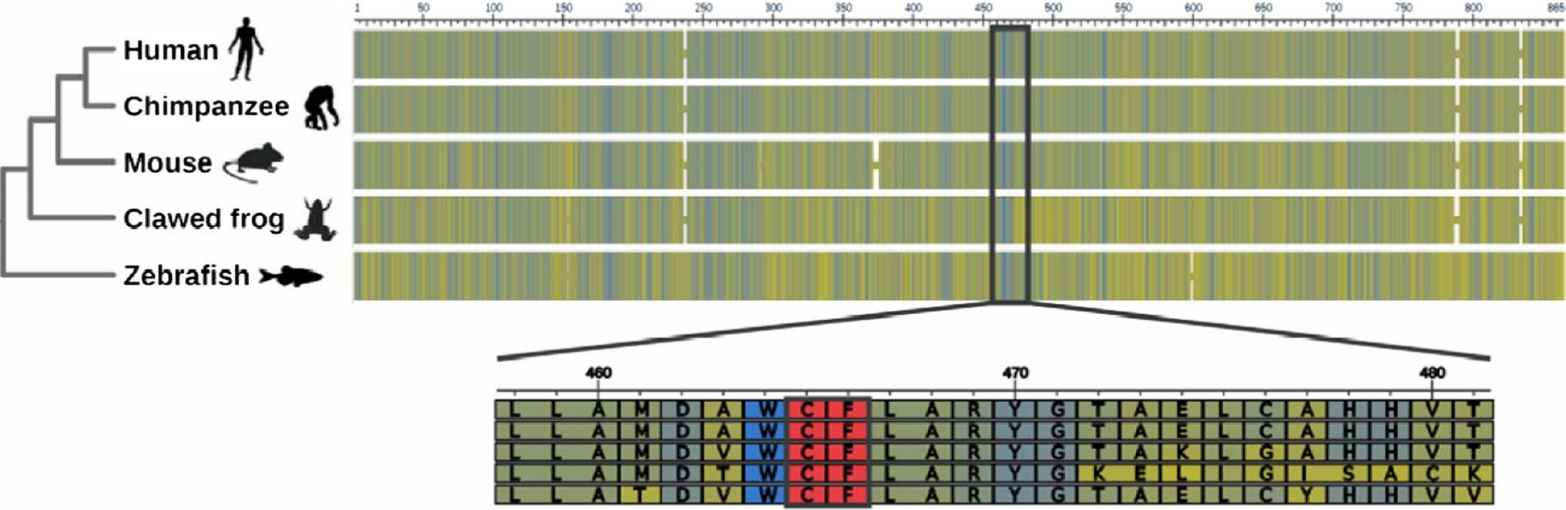
C1ORF112 alignment in five selected species. The protein alignment was downloaded from the OMA genome browser [21] showing a high level of conservation among vertebrates. We used BLOSUM62 (Blocks Substitution Matrix) to identify similarities arising between the species [22]. The sequence in blue represents a threshold of 62% identity, and the red positions represent the WCF conserved amino acids

Chromosome 1 Open Reading Frame 112 (C1ORF112) codes for nine transcripts. The chromosomal locus for C1ORF112 is 1q24.2. It can also be identified as FLJ10706 (HGNC) or ENSG00000000460 (Ensembl). C1ORF112 is encoded in a 5′ → 3′ forward direction. Of nine transcripts, five are translated into proteins, while four undergo nonsense‐mediated decay. The first two transcripts are 853 amino acids in length and comprise 4355 bps containing 24 exons and 4011 bps containing 25 exons, respectively [11]. There are no domain motifs currently attributed to this protein, as it is currently classified under the domain of unknown function DUF4487 [12]. Proteins in this domain family have a conserved WCF tripeptide sequence (Fig. 1) which may be of functional relevance [12].

### Evolutionary history of C1ORF112

We reconstructed the evolutionary history of *C1ORF112* using 67 orthologous sequences from representative species of all major groups of Eukaryotes. The orthologues were sought within the Orthologous Matrix project (OMA) [13], using the Pfam entry [14], and entry DUF4487 and also through local BLAST searches [15, 16]. Protein sequences were aligned using the L‐INS‐I strategy from MAFFT v747 [17]. We inferred the gene tree of *C1ORF112* using the maximum-likelihood program IQ‐TREE multicore version 1.6. for Linux [18]. The best model of substitution (JTT + F + G4) was selected using ultrafast bootstrap replicates [19]. iTOL v5.5.1 [20] was used for gene tree visualisation and the images were obtained from PhyloPic (http://phylopic.org/). The multiple sequence alignments show high levels of conservation within the gene sequences. As stated earlier, there is a conserved WCF tripeptide in most of the species, and this is usually preceded by the amino acid LAMDA, followed by the amino acids LARY, predominantly in vertebrate sequences. Sequence alignment of model organisms in which the gene is present indicates that the WCF amino consistently present (Fig. 1).

**Fig. 2:**
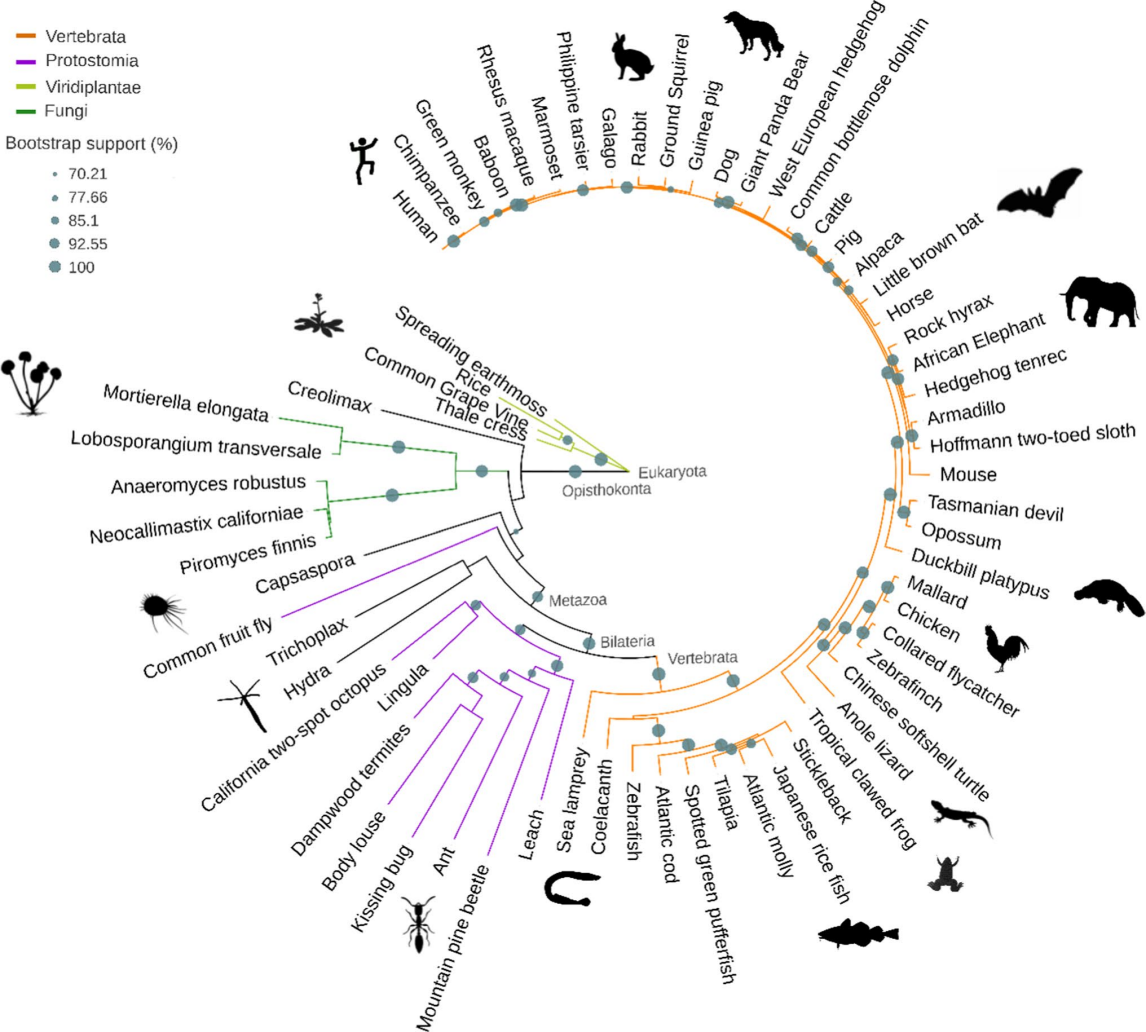
C1ORF112 gene tree. The phylogenetic relationship arising between the main groups of Eukaryota and different species are in general well conserved from plants to humans, with a bootstrap support > 70%

We found that *C1ORF112* may have originated in the ancestor of all Eukaryotes (Fig. 2). The phylogenetic relationship between the main groups (e.g., vertebrates, protostomia, viridiplantae, and fungi), as well as between species are, in general, well conserved from plants to humans. However, notable exceptions are found; for example, *Drosophila* falls outside the Metazoa and the mouse falls into the sister group to placental mammals (Fig. 2). In addition, most organisms containing a homologue of C1ORF112 are multi‐cellular organisms with notable exceptions such as *Acanthamoeba castellanii, Capsaspora spp*, and *Creolimax fragrantissima*. C1ORF112 homologs are found in plants, but appear to be mostly absent in fungi, suggestive of a gene loss event, exceptions being the early diverging fungal group *Chytridiomycota* and the *Mucoromycota*. Sensitive HMM–HMM searches at the HHpred server [23] were used to further query model organisms in which homologs appear to be absent. These searches confirmed the absence of C1ORF112 proteins in *S. cerevisiae* and *C. elegans*, but revealed *CG13742* to be the likely *D. melanogaster* homolog (Table 1). However, *CG13742* does not possess the WCF tripeptide, which is present in other model organisms, and the sequence identity is also quite low when compared to the sequences of other model organisms (Table 1). Overall, *C1ORF112* is evolutionarily well‐conserved across vertebrates, with homologues also present in some invertebrates, plants, and single‐celled microorganisms.

**Table 1:** C1ORF112 amino acid sequence similarity between humans and selected biomedical model organisms

Species	Amino acid % similarity with *Homo sapiens* C1ORF112 (Gene ID: 55732; Ensembl: ENSG00000000460)
*Mus musculus* (Entrez Gene ID: 381306; Ensembl: ENSMUSG00000041406)	67.7%
*Rattus norvegicus* (Entrez Gene ID: 498265; Ensembl: ENSRNOG00000059276)	69.0%
*Danio rerio* (Entrez Gene ID: 553598; Ensembl: ENSDARG00000042120)	42.8%
*Drosophila melanogaster* (Entrez Gene ID: 35916; FlyBase: FBgn0033372)	11.8%
*Caenorhabditis elegans*	No homologs found
*Saccharomyces cerevisiae*	No homologs found

### Structure of the C1ORF112 protein

The Pfam entry corresponding to C1ORF112 and DUF4487 records no structures for family members. Indeed, BLAST searches of the Protein Data Bank [15, 24] yield no significant hits, demonstrating that no close homologues of *C1ORF112* have yet been structurally characterised. Nevertheless, the sensitive HMM–HMM comparison method HHpred [23] revealed significant matches to proteins with repetitive alpha helix‐rich structures. The strongest match, with a probability of 87% (albeit with a sequence identity of only 8%), was to the beta subunit of the human importin which contains HEAT repeats (e.g., PDB code 1qgr [25]). Other proteins containing HEAT repeats, such as microtubule‐ binding TOG domains (e.g., 2 of 3 [26]), PTPA protein phosphatase activator (e.g., 4 lac [27]), and the yeast cytoplasmic export protein 1 (e.g., 3vwa [28]) also achieved significant scores.

**Fig. 3:**
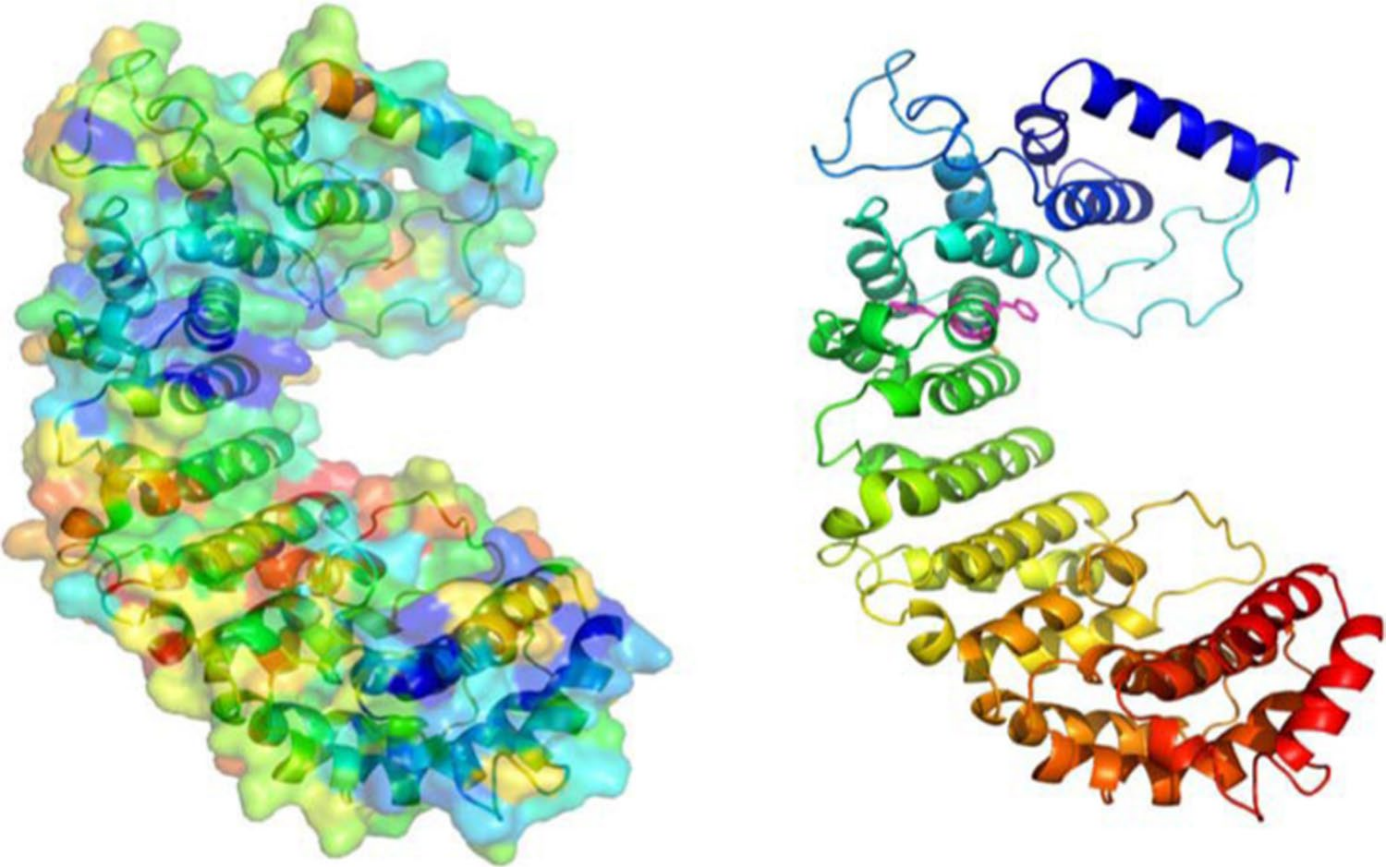
3D structure of C1ORF112. An approximate structural model of residues 304–763 of C1ORF112 based on the alignment produced by HHpred [23] with importin beta subunit (1qkg;). Conservation mapping with the ConSurf server [31] left (blue indicates high conservation in the C1ORF112 family; red indicates low conservation) reveals two conserved patches, the upper containing the conserved WCF motif (magenta sticks on the right), where the protein is coloured blue to red from the N‐ to the C‐terminus

HEAT repeats, and the related ARM repeats, form part of a large superfamily of repetitive structures in which the repeating unit, around 50 residues long, contains two or three helices [29]. Despite their involvement in a wide variety of cellular processes, they share a molecular mechanism in which the repeats trace out a curved 3D structure on the inner side in which residues from multiple adjacent repeats combine to form protein–protein interaction sites. Using the HHpred alignment between C1ORF112 and the importin beta subunit, a molecular model of residues 304‐763 of C1ORF112 was constructed using the Swiss Model server [30]. The low-sequence identity and high evolutionary distance arising between *C1ORF112* and the importin beta subunit ensure that the model quality will be limited. Nevertheless, the ConSurf server [31], when used for mapping sequence conservation in the C1ORF122 family onto the approximate model, revealed that the inner side of the curved structure is significantly more conserved than the outer surface (Fig. 3). This provides independent support for the expectation that the inner surface harbours interfaces for binding to its interaction partners. The model also illustrates the structural context of the aforementioned WCF motif: the Trp residues form part of the hydrophobic core, whereas the Phe residue lies on the surface where it is likely to contribute to interactions with a partner protein. Interestingly, the model of the central portion of C1ORF112 reveals two significant conserved surface patches, one containing the WCF motif and the other located towards the end of the modelled region. These may function as distinct interfaces for different sets of interactors, but could also, as in the importin beta subunit [32], target distinct regions of the same large interactor. We also validated the above results by modelling C1ORF112 in another well‐known server called I‐TASSER (Zhang 2008), one which uses threading and ab‐initio methods to predict the structure and found similar folding patterns.

### Putative functions of C1ORF112

#### Early studies linking C1ORF112 to cancer

The exact functions of C1ORF112 in humans and other vertebrates are currently unknown. However, there have been several studies that afford insights into its potential actions. Leo et al. (2005) studied the gene regulation profile of hormone‐independent breast cancer cells which had been transfected with the progesterone receptor (PR), but were otherwise negative for the oestrogen receptor (ER‐). The results of this study revealed a threefold down‐regulation of C1ORF112 (known at the time as FLJ10706) in the presence of PR [10], suggesting that C1ORF112 could be a progesterone-regulated gene. Another study [9] focused on understanding genomic and proteomic profiles of gelsolin in relation to TP53 status and bladder cancer progression. These showed a twofold down-regulation of C1ORF112, placing it in the top 30 differentially expressed genes arising between wild‐type presenting TP53 and mutant TP53 cells within invasive bladder tumour cells. C1ORF112 was also found to have a twofold up‐regulation in desmoid tumours when compared to normal fibroblasts [33]. Another regulator implicated in acting upon C1ORF112 is guanosine‐5′‐triphosphate (GTP); a well‐known G‐protein modulator, which was shown to effect a > two fold up‐regulation of C1ORF112 in SH‐SY5Y cells. However, the mode of action and the implications of this interaction is yet unknown [34]. Each of these independent studies, including the earlier mentioned van Dam et al. study [3], indicates that C1ORF112 must interact with proteins involved in cell‐cycle progression and suggest possible interactions with tumorigenic genes.

#### Mouse studies suggesting a role for BC055324 in early development

Interestingly, knockout mice of the *C1ORF112* homologue, *BC055324* are embryonically lethal (https://www.mousephenotype.org/data/genes/MGI:3590554), suggesting that *BC055324* plays an important function, at least during early development [35]. Microarray data from the Genevestigator database (using the Mouse Genome 430A 2.0 Array) show that *BC055324* is very highly expressed in the renal vesicles, mesenchyme, ureteric bud, embryonic cells, blastocyst cells, oocytes, and foetal haemopoietic stem cells [36], again strongly suggesting a role in early development. Further evidence to support this argument comes from the effect of deleterious mutations of the transcriptional enhancer *M1442* in generating significant reductions in transcriptional variation in *BC055324*, bringing the gene physically closer to regulatory elements which confer transcriptional robustness [37, 38]. As genes which play a significant role in development often have tightly controlled transcriptional regulators, this is further evidence for a role of BC055324 in development which is tightly regulated by *M1442* (a homolog of human *H1442*).

While homozygous animals do not survive, heterozygous animals have been phenotyped as part of the International Mouse Phenotyping Consortium [35, 39]. Briefly, heterozygous animals have been reported to show an increase in lean body mass and circulating cholesterol levels, as well as abnormal bone mineralization, possibly resulting in decreased bone mineral density (https://www.mousephenotype.org/data/genes/MGI:3590554). Mouse models are available for BC055324 that permit researchers to conduct further such studies [35]. Unpublished work by Cordeiro, Edogbanya, and de Magalhães et al. suggests that the ablation of *BC055324* in adult animals using conditional knockout mice yields no obvious phenotypic differences.

#### Functional insights from co‐expression and protein interactions

As aforementioned, *C1ORF112* was initially found to be strongly co‐expressed with cancer‐related genes [3]. To be more precise, mouse genes significantly co‐expressed with a seed list of previously published cancer‐associated genes were, based on a “guilt‐by‐association” approach, used to identify novel cancer‐associated candidate genes [3]. Among the candidate cancer‐associated genes were some poorly studied genes, the most statistically significant of which was *BC055324*. The human homolog of *BC055324* is *C1ORF112*. Like *BC055324*, *C1ORF112* is co‐expressed with several genes associated with cancer and is strongly co‐expressed with *BRCA1* and *BRCA2* genes [3]. In addition, genes that play important roles in cell cycle regulation and cancer formation are among the most strongly co‐expressed with *C1ORF112*. Silencing C1ORF112 reduces growth of the HeLa cancer cell line when compared to control siRNAs [3].

Querying C1ORF112 in STRING [40] reveals several text-mining gene associations from van Dam et al. (2012), but also strong links via gene co‐expression between C1ORF112 and ten proteins. The strongest three scores are for ASPM, CENPF, and NCAPG, proteins linked in various ways to mitosis. Indeed, when the set of C1ORF112 and the ten associated matches are tested for statistical enrichment of terms in standard databases and ontologies, the cell cycle is the most significant Biological Process in the Gene Ontology (false discovery rate FDR, 4.9e‐07) while, for UniProt [41], mitosis is the keyword most strongly linked (FDR, 1.7e‐07). A more recent analysis of the expression of stem cell-related genes in gastric cancer revealed a co-expression module of 16 genes associated with stem cell self-renewal and cell proliferation, one of which was C1ORF112 [42]; another similar study also found C1ORF112 as part of a nine-gene risk model associated with disease outcomes in gastric cancer patients [43]. These findings confirm that the co‐expression‐based implications of a potential involvement of C1ORF112 in cell cycle and cancer hold when the large increase in expression data obtained since its initial discovery in 2012 are considered.

**Fig. 4:**
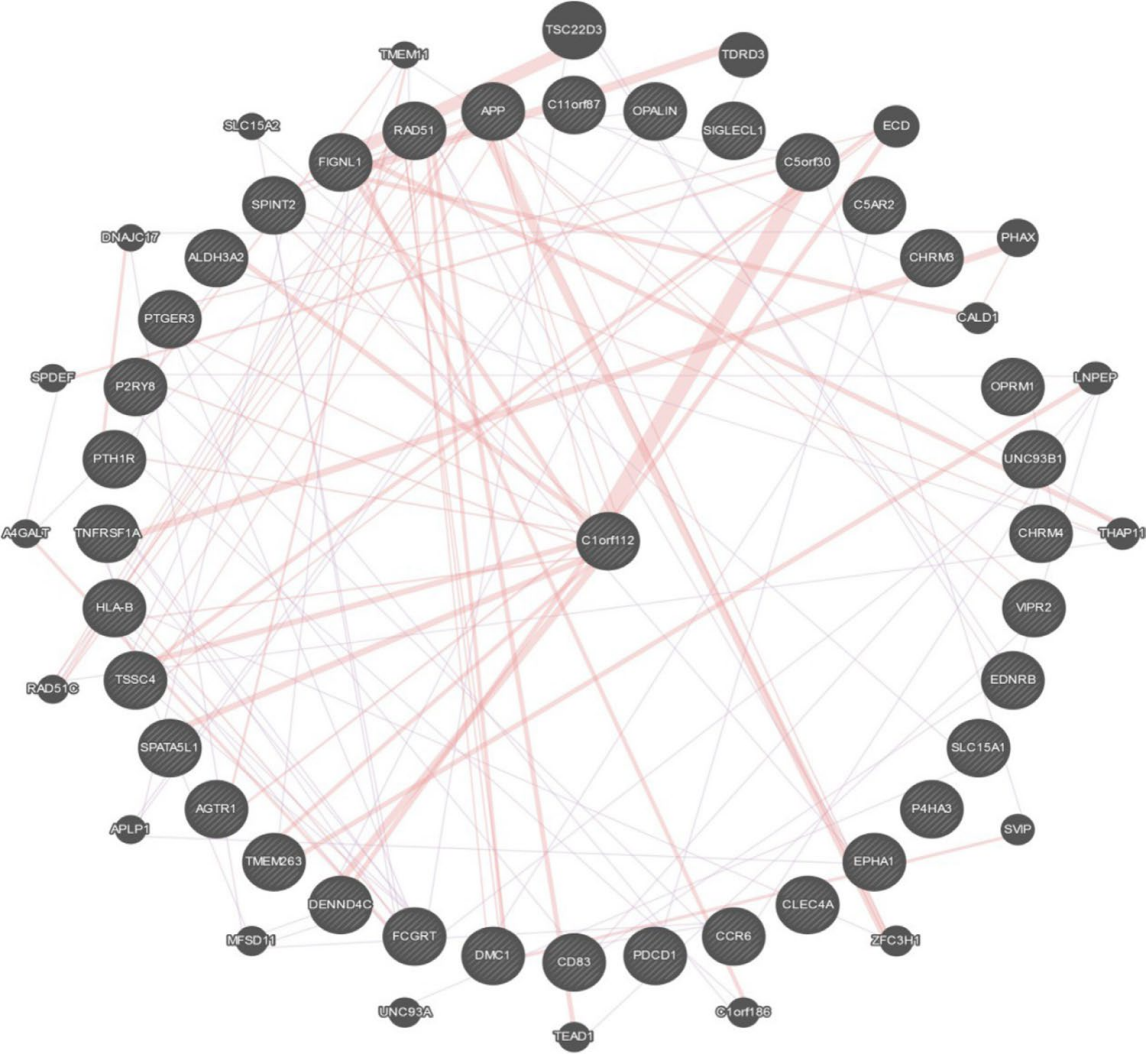
Potential protein–protein interactions with C1ORF112 (centre) obtained through BioGRID [44]. Figure rendered using the GeneMANIA website [45]. Pink lines indicate possible physical interactions, while blue ones indicate co‐expression interactions

Furthermore, whether acting as bait or hit interactors, various high-throughput affinity capture mass spectrometry (MS) experiments obtained from the BioGRID database [44] reveal 31 physical protein–protein interactions for C1ORF112 (https://thebiogrid.org/120851/summary/homo‐ sapiens/c1orf112.html). These interactions are shown in Fig. 4.

#### Proteomic studies and functions of C1ORF112 in DNA repair and the Fanconi anaemia pathway

Recent proteomic studies on the repair of DNA inter‐strand crosslinks (ICL) observed *C1ORF112* to be recruited to protein complexes at ICL-stalled DNA replication forks in *Xenopus* egg extracts [46]. The stalling of DNA replication forks arises due to ICLs recruiting around ninety DNA repair and genome maintenance factors. These included FA pathway proteins that coordinate the repair of ICLs, homologous recombination repair proteins, including BRCA1 (FANCS), and BRCA2 (FANCD1), the SLF1/SLF2 protein complex, and C1ORF112. This strongly indicates that C1ORF112 might serve a role in DNA repair, specifically in responses to replication stress brought about by DNA damage through ICL [46]. These various findings strongly implicate a role for C1ORF112 in DNA replication.

A role for C1ORF112 in DNA damage responses is further indicated by the observation that C1ORF112 ‐/‐ cells exhibit a significant hypersensitivity to mitomycin C (MMC), a canonical inducer of ICLs. ICL‐hypersensitivity is a hallmark of both FA‐ and HRR‐defective cell lines and, further, C1ORF112 ‐/‐ cells additionally display other FA‐like features (Bell and Jones, manuscript in preparation). These include MMC-induced chromosomal aberrations, including chromatid abnormalities and radial rearrangements, notwithstanding a G2/M cell cycle delay, all of which are characteristics of FA‐defective cells [47]. These observations indicate a likely role for C1ORF12 within the FA pathway itself or in its regulation.

Cells that are defective in HRR, including BRCA1 and BRCA2/FANCD1, in addition to other components of the downstream FA pathway, are typically hypersensitive to DNA damaging agents other than ICLs [48]. These include mono‐functional alkylating agents such as methyl methanesulphonate (MMS), ethyl methanesulphonate (EMS), and methylnitronitrosoguanidine (MNNG), all of which can give rise to DNA replication fork stalling and indirect DNA double‐strand breaks. C1ORF112 knockout cells exhibit significant hypersensitivity to MMS and EMS and express the mono-ubiquitylated form of FANCD2, suggesting that, if C1ORF112 does function within the FA pathway, it is likely to be in the downstream HRR part of the pathway.

Significantly, one recent study, using RPE1‐hTERT Cas9 *TP53 − / − *cells to perform genome‐scale CRISPR screens against DNA‐damaging agents, found C1ORF112 to be important in resistance to DNA‐damaging agents, including Cisplatin‐2, Cisplatin‐3, and MNNG [49]. Importantly, C1ORF112 very strongly clusters with FA/ICL repair genes within the map constructed for this study [49]. The sensitivity to cisplatin drugs (ICL‐inducing) and MNNG is consistent with the sensitivities of C1ORF112 ‐/‐ and further alludes to a functional association of C1ORF112 within the FA pathway.

Intriguingly, the pivotal FA pathway protein FANCD2 has very recently been demonstrated to directly interact with ALDH3A2 (Wataru Sakai, Kobe University Japan, personal communication), which itself directly interacts with C1ORF112 (see Fig. 4). ALDH3A2 is an aldehyde dehydrogenase that catalyses the oxidation of medium and long-chain aliphatic aldehydes to fatty acids [50]. A major function of the FA pathway and FANCD2, specifically, is in mediating the DNA damage response to the toxic effects of endogenous aldehydes [51]. The interaction with both FANCD2 and ALDH3A2 intimates a similar function for the C1ORF112 protein.

Some FA proteins, including BRCA2 (FANCD1), are known to play an important role in the formation of chiasmata and meiotic segregation in mammalian cells [52]. A possible role for C1ORF112 in meiosis has also been demonstrated in plants. There have been studies of plant homologues of *C1ORF112*, a homology that was inferred from the presence of the sequences in DUF4487, and these sequences contain the WCF tripeptide. One of these studies is of a novel rice (*Oryza sativa*) protein MEICA1, which contains the conserved WCF tripeptide and is present within the domain DUF4487. MEICA1 is suggested to interact with TOP3α and thereby regulate meiotic segregation [53]. This interaction enabled accurate meiotic segregation in the pachytene stage of wild‐type seeds. Mutant seeds, although showing normal vegetative growth, were mostly sterile due to an aberrant association of non‐homologous chromosomes [53]. Another study of the rockcress (*Arabidopsis thaliana*) protein AT1G04650 (also known as Holliday junction resolvase or FLIP) which similarly contains this conserved WCF tripeptide sequence and is present within the domain DUF4487 was identified via tandem affinity purification mass spectrometry as a meiotic crossover limiting factor [8]. AT1G04650 is reported to act in tandem alongside FIGL1 to limit meiotic crossover. Conversely, *A. thaliana* FANCD2 promotes meiotic crossover formation [54], hinting at yet another functional link between C1ORF112 and the FA pathway.

#### Tissue expression and regulatory associations of C1ORF112

**Fig. 5:**
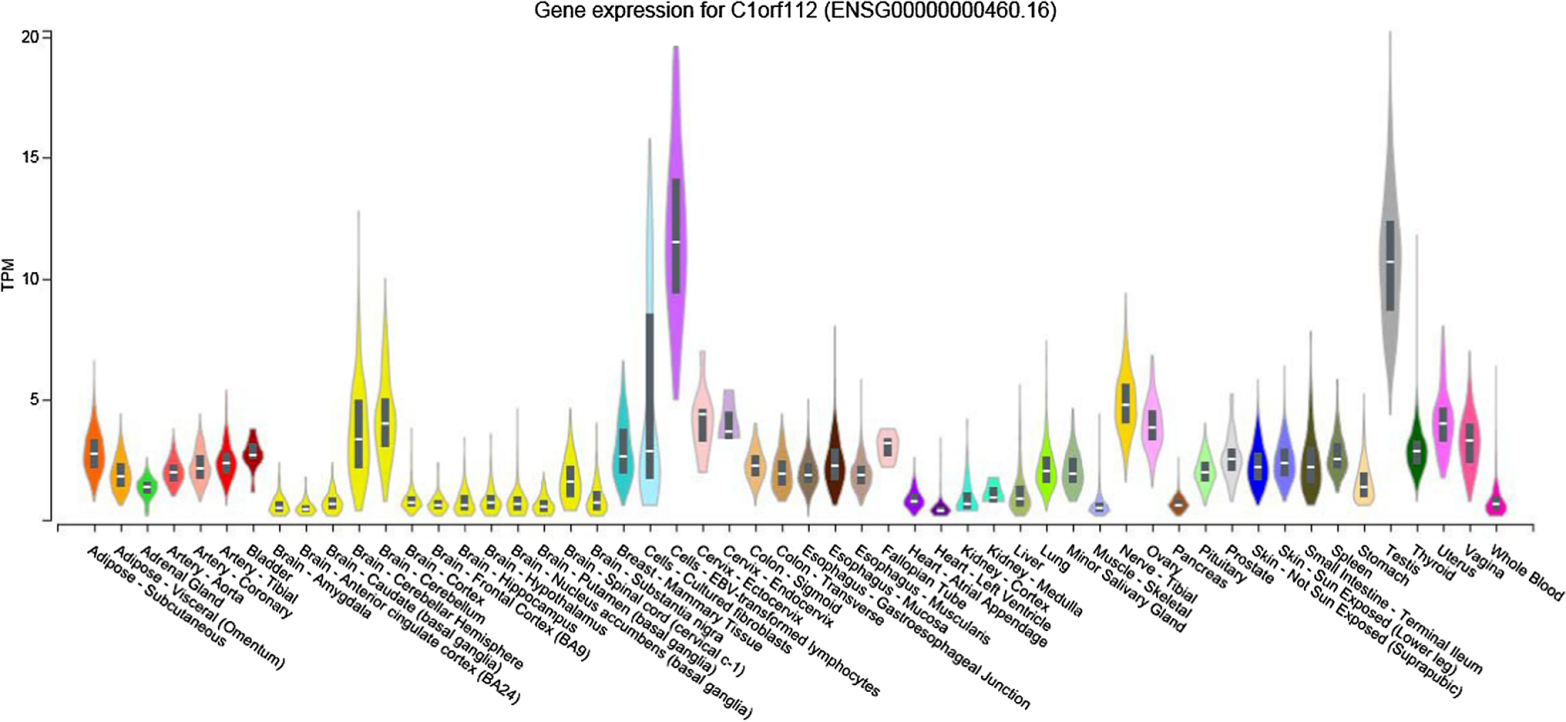
Relative levels of expression of C1ORF112 across various cell and tissue types within the body. C1ORF112 shows relatively higher levels of expression in the testis and in Epstein–Barr virus‐transformed lymphocytes. Data obtained from the GTEx portal (https://gtexportal.org/home/gene/C1ORF112)

Due to the paucity of information relating to C1ORF112 within the extant literature, we searched other online resources to gain insights into the regulation and possible functions of C1ORF112. One fluorescent antibody (obtained from the Protein Atlas (https://www.proteinatlas.org/ENSG00000000460‐C1orf112/) suggests that C1ORF112 may co‐localise to the mitochondria, although this assertion needs to be further validated. Furthermore, the Protein Atlas showed that C1ORF112 is widely expressed in a number of cell types with no significant tissue specificity. Expression of the C1ORF112 RNA has been detected in Karpas‐707 cell lines derived from lymphoid cells, and also in K‐562-derived from myeloid cells with a high level of RNA expression which is some 22-to-25-fold higher than observed in other cell lines [55]. Cells with lymphoid and myeloid lineages, such as monocytes, basophils, and natural killer cells, exhibit higher levels of expression of C1ORF112 RNA, which are > fourfold higher than measured in other cells [55]. C1ORF112 is also expressed in many tissues. However, it is should be noted that it exhibits a very high expression in endocrine tissues such as the testis and parathyroid gland, as well as in primary lymphoid organs like the thymus [56]. These results align with other data, including that of the BioGPS [57], which indicates that the testis has a higher level of expression of C1ORF112 as compared to other organs (http://biogps.org/#goto=genereport&id=55732). Likewise, according to the GTEx portal [58], C1ORF112 exhibits high levels of expression within the testis and also in Epstein–Barr virus‐transformed lymphocytes, although there is only a modest level of expression in the brain and a relatively low level of expression in most other cells and tissues within the body (Fig. 5).

ChIP‐seq data from the ENCODE database (Consortium 2004) show elevated levels of active H3K27Ac marks present within the promoter region of C1ORF112. Various cell lines, ranging from cancer to embryonic stem cells, exhibit higher chromatin accessibility which indicates that this gene is highly accessible to epigenetic acetylation marks. Furthermore, the ENCODE database reveals significant transcription levels of C1ORF112 in several cell types according to RNA‐seq. The GM12828, H1‐hESC, HeLa‐S3, HepG2, and K562 cell lines show the highest levels of transcription of C1ORF112. Many regulatory regions tend to be DNase‐sensitive, as the open chromatins are easily cleaved by DNase. The same phenomenon is observed within the promoter region of C1ORF112, suggesting that it has the open chromatin needed for its expression. C1ORF112 possesses a well‐conserved active promoter region that is featured across many different cell lines.

Finally, the GWAS catalogue [59] reveals potential associations of C1ORF112 with amyotrophic lateral sclerosis, acne, epigenetic age acceleration, venous thromboembolism, and blood protein levels (https://www.ebi.ac.uk/gwas/genes/C1orf112), and these findings open further avenues for future studies.

### Potential relevance of C1ORF112 to cancer

Data from various databases such as BioGPS and GTEx reveal that C1ORF112 has a strong correlation with oncogenesis through co-expression with genes previously related to cancer. C1ORF112 is highly expressed within both breast and cervical cancer. Furthermore, it is expressed at high levels within the testis in both normal and cancerous cells. For example, *C1ORF112* is generally overexpressed in neoplasia derived from various organs when compared to healthy tissues [60]. Similar results have been obtained from Genevestigator data [36], which revealed high levels of expression of *C1ORF112* across various tumours. A gain in copy number in *C1ORF112* has also been observed in some types of cancers and, most significantly, in breast cancer [61, 62]. Furthermore, expression levels of C1ORF112 are correlated with survival in endometrial cancer, and patients with higher levels of expression of the gene also have a poorer prognosis (https://www.proteinatlas.org/ENSG00000000460‐C1orf112/pathology). Therefore, further studies of the C1ORF112 gene and its various protein forms are certainly warranted. *C1ORF112* could also present a potential therapeutic target and serve as a diagnostic marker.

C1ORF112 is also linked with head and neck cancers, such as head and neck squamous cell carcinoma (HNSCC), according to Genevestigator [36], and it is significantly overexpressed in both these forms of cancer [63]. Interestingly, HNSCC is extremely common in Fanconi anaemia patients [4]. Given that this susceptibility to HNSCC results from a defect in the FA pathway, it is tempting to speculate that overexpression of C1ORF112 leads to a dysregulation of the FA pathway which may, in turn, contribute to the development of HNSCC in non‐FA individuals. Clearly, further investigations are required to confirm any definitive association with either FA or HNSCC.

## Conclusions

Given the expression patterns and identified interactions of C1ORF112, it could well be a DNA replication‐associated protein. If this theory is confirmed, it might be a useful candidate protein to study in the context of cell cycle regulation, DNA damage responses, homologous recombination, maintenance of genome integrity, and, ultimately, cancer progression. C1ORF112 is potentially an FA‐associated gene, although it is highly conserved from an evolutionary standpoint, unlike most of the upstream FA genes. However, given its strong associations with both Fanconi anaemia and homologous recombination repair, and the observation that it is embryonically lethal (like BRCA1 and BRCA2), this places C1ORF112 in the downstream stretch of the FA pathway. The prevailing evidence indicates an intimate association with the cell cycle as well as the DNA repair of double‐strand breaks and stalled replications forks that are associated with inter‐strand crosslinks and chromosomal segregation. It might be hypothesised that C1ORF112 is a nuclear protein, one which is involved in the efficient coupling and uncoupling of DNA strands during homologous recombination repair and chromosomal segregation in meiotic cells.

In summary, the evidence to date reveals that C1ORF112 is essential for normal mammalian development and has important biological functions, as is evidenced from data obtained from both mice and mammalian cells. Still, there seems to be more in the way of questions than answers in relation to the function of C1ORF112. Clearly, much more work is needed to understand the potential roles of C1ORF112 in cell cycle regulation, maintaining genomic stability and normative DNA damage signalling pathways, not to mention a future exploration of its potential associations with oncogenesis. A deeper understanding of C1ORF112’s cellular mechanics is required to determine the nature of its intimate associations with the regulation of the cell cycle as well as in DNA replication and repair. This knowledge will ultimately determine whether its modulation might be beneficial in regulating cancer and its outcomes.
